# Redox Imbalance and Mitochondrial Abnormalities in Kidney Disease

**DOI:** 10.3390/biom12030476

**Published:** 2022-03-21

**Authors:** Liang-Jun Yan

**Affiliations:** Department of Pharmaceutical Sciences, College of Pharmacy, University of North Texas Health Science Center, Fort Worth, TX 76107, USA; liang-jun.yan@unthsc.edu; Tel.: +1-817-735-2386; Fax: +1-817-735-2603

The kidneys carry out fundamental life-sustaining functions by removing waste substances, controlling salt and water balance, retaining substances vital to the body such as glucose and proteins, and maintaining blood pH balance [1]. By performing these functions, the kidneys are heavily dependent on mitochondrial ATP production that not only consumes a large amount of oxygen, but also exposes each kidney to a variety of damages, such as diabetic hyperglycemia, drug toxicity, ischemic injury, heavy metal toxicity, and sepsis [2]. All these damages are thought to be associated with cellular redox imbalance and mitochondrial oxidative stress, as well as mitochondrial abnormalities. The clinical symptoms of these insults can be anywhere from acute kidney injury (AKI) to chronic kidney disease (CKD) that, if left untreated, can eventually lead to end-stage kidney failure [3]. 

To fend off the abovementioned insults, it is necessary to understand the underlying mechanisms of redox imbalance, oxidative stress, and mitochondrial abnormalities involved in kidney disease. In this Special Issue of *Biomolecules* entitled “Redox Imbalance and Mitochondrial Abnormalities in Kidney Disease”, we have collected 11 papers that cover a variety of topics focusing on oxidative stress, mitochondrial dysfunction, and antioxidation enhancement implicated in kidney disease or kidney transplantation. 

Among the 11 papers, seven are review articles, and four are original research articles. Yan reviewed the sources of NAD^+^/NADH redox imbalance and their roles in diabetic kidney disease (DKD) [3]. The gist of the paper is that NAD^+^/NADH redox imbalance leads to the disruption of mitochondrial homeostasis, and an increase in nephron oxidative damage that culminates as diabetic nephropathy reflected by deteriorating kidney functions. While mitochondrial oxidative stress is a central event in DKD, it is also hypothesized by Yan and Allen to be a unifying mechanism underlying cadmium-induced kidney toxicity [2] that mainly involves oxidative damage to proximal tubule epithelial cells within the nephrons. Moreover, Zhu et al. also stressed in their review article that mitochondrial oxidative stress-induced cell death is the major mechanism of podocytopathies involving the dysregulation of p53, PI3K/Akt, P38/MAPK, unfolded protein response, and endoplasmic reticulum that may all be the downstream targets of oxidative stress and redox imbalance [4]. 

In the article “Glucose- and non-glucose-induced mitochondrial dysfunction in diabetic kidney disease” [5], Ito et al. summarized the effects of toxic metabolites of glucose, such as advanced glycation end-products and the detrimental effects of non-glucose events, such as lipotoxicity, hypoxia, and endothelin-1 receptor signaling on the pathogenesis of DKD [4]. It should be noted that lipotoxicity can also be caused by the accumulation of acetyl-CoA, which is not only a substrate for lipid biosynthesis, but can also lead to an increase in protein acetylation [6]. Both processes are thought to contribute to the pathogenesis of kidney disease, along with the potential contributions of Krebs cycle metabolites, such as citrate, succinate, and fumarate [6]. 

Given that mitochondrial redox imbalance and oxidative stress are the central themes of kidney disease, it is natural to explore therapeutic approaches that can counteract kidney injury by mitigating oxidative stress and mitochondrial dysfunction. Indeed, several papers in this Special Issue have just explored such approaches. For example, the role of SGLT2 inhibitors as antioxidants in the kidney was reviewed by Liorens-Cebria et al. [7]. Additionally, Wongmekiat et al. provided evidence that purple rice husk is nephoprotective in DKD, with an underlying mechanism involving PGC-1α, Sirt3, and SOD2 [8]. Interestingly, renalase, as a flavin-dependent detoxifying enzyme, may be regarded as a risk factor for death in patients with CKD [9], though further studies are needed to confirm the link between renalase levels and death. Approaches that can prevent mitochondrial uncoupling and preserve renal function in transplanted kidneys have also been discussed in this Special Issue. These include the controlled oxygenated rewarming of kidney grafts [10] and the suppression of inflammation-induced kidney damage by the use of a free radical scavenger Prc-210 [11]. The underlying protective mechanisms of these approaches likely involve the mitigation of mitochondrial oxidative stress and the restoration of redox balance.

In summary, mitochondrial oxidative stress and redox imbalance drive renal mitochondrial abnormalities, which can be attributed to the over-production of mitochondrial reactive oxygen species. As summarized by Aranda-Rivera et al. and Ito et al. in their review articles published in this Special Issue [5,12], numerous processes and signaling pathways are involved, and these can collectively contribute to the pathogenesis of a given kidney disease. These pathways include posttranslational modifications of proteins, mitochondrial fusion and fission, mitophagy, biogenesis, Warburg effects, Krebs cycle, electron transport chain, oxidative phosphorylation, and mitochondrial uncoupling. Further dissection of each of these processes or signaling pathways may provide novel insights into designing additional therapeutic approaches to fight kidney disease.
